# Cardiac responses to viewing facial emotion differentiate frontotemporal dementias

**DOI:** 10.1002/acn3.563

**Published:** 2018-04-14

**Authors:** Charles R. Marshall, Christopher J. D. Hardy, Micah Allen, Lucy L. Russell, Camilla N. Clark, Rebecca L. Bond, Katrina M. Dick, Emilie V. Brotherhood, Jonathan D. Rohrer, James M. Kilner, Jason D. Warren

**Affiliations:** ^1^ Dementia Research Centre Department of Neurodegenerative Disease Institute of Neurology University College London Queen Square London WC1N 3BG UK; ^2^ Sobell Department of Motor Neuroscience and Movement Disorders Institute of Neurology University College London Queen Square London WC1N 3BG UK; ^3^ Wellcome Trust Centre for Neuroimaging Institute of Neurology University College London Queen Square London WC1N 3BG UK

## Abstract

**Objective:**

To establish proof‐of‐principle for the use of heart rate responses as objective measures of degraded emotional reactivity across the frontotemporal dementia spectrum, and to demonstrate specific relationships between cardiac autonomic responses and anatomical patterns of neurodegeneration.

**Methods:**

Thirty‐two patients representing all major frontotemporal dementia syndromes and 19 healthy older controls performed an emotion recognition task, viewing dynamic, naturalistic videos of facial emotions while ECG was recorded. Cardiac reactivity was indexed as the increase in interbeat interval at the onset of facial emotions. Gray matter associations of emotional reactivity were assessed using voxel‐based morphometry of patients’ brain MR images.

**Results:**

Relative to healthy controls, all patient groups had impaired emotion identification, whereas cardiac reactivity was attenuated in those groups with predominant fronto‐insular atrophy (behavioral variant frontotemporal dementia and nonfluent primary progressive aphasia), but preserved in syndromes focused on the anterior temporal lobes (right temporal variant frontotemporal dementia and semantic variant primary progressive aphasia). Impaired cardiac reactivity correlated with gray matter atrophy in a fronto‐cingulo‐insular network that overlapped correlates of cognitive emotion processing.

**Interpretation:**

Autonomic indices of emotional reactivity dissociate from emotion categorization ability, stratifying frontotemporal dementia syndromes and showing promise as novel biomarkers. Attenuated cardiac responses to the emotions of others suggest a core pathophysiological mechanism for emotional blunting and degraded interpersonal reactivity in these diseases.

## Introduction

Frontotemporal dementia (FTD) comprises a spectrum of neurodegenerative disorders with three major syndromes^1^; behavioral variant (bvFTD), semantic variant primary progressive aphasia (svPPA), and nonfluent variant primary progressive aphasia (nfvPPA). This classification admits considerable heterogeneity; in particular, bvFTD comprises several clinico‐anatomical subsyndromes, of which the most distinctive is the variant with predominant right temporal lobe atrophy (right temporal variant; rtvFTD).^2^, ^3^ Deficits in emotion processing and empathy are prominent in all FTD syndromes,^4^, ^5^ but remain poorly characterized and difficult to quantify. Conventional neuropsychological instruments emphasize the cognitive categorization of emotions, which is potentially confounded by coexisting semantic deficits. Moreover, emotion labeling tasks do not capture the dynamic emotional reactivity that is central to interpersonal functioning in daily life.^6^

In health, responding to others’ emotions comprises both cognitive and affective components, which are dissociable and have distinct anatomical bases.^7^ Central to understanding affective empathy is the concept of interoceptive inference, which proposes that emotional awareness entails reciprocal feedback between somatic physiology and the cognitive interpretation of those signals.^8^, ^9^ Emotional stimuli produce autonomic effects including modulation of heart rate, but different emotions do not reliably produce specific individual patterns of autonomic responses, and they are therefore hypothesized to relate to arousal and intensity rather than emotion category.^10^, ^11^ Stimulus onset induces a cardiac orienting deceleration, which is modulated by affective content, with greater cardiac deceleration accompanying higher emotional valence.^12^, ^13^, ^14^ This central regulation of cardiac function is mediated by a distributed brain network including anterior cingulate cortex (ACC), insula, and orbitofrontal cortex (OFC).^15^, ^16^ Cardiac afferent information informs affective valuation,^17^ and visceral autonomic responses may support emotional contagion and empathy.^9^

If autonomic mechanisms contribute to aberrant emotion processing in FTD, one would anticipate associated changes in physiological reactivity, as has previously been documented in FTD syndromes. In particular, bvFTD has been associated with abnormal autonomic reactivity to affectively charged stimuli,^18^, ^19^, ^20^, ^21^, ^22^ alterations of resting skin conductance and heart rate variability,^23^, ^24^ and abnormal brain‐heart coupling,^24^, ^25^ while nfvPPA has been associated with reduced pupil responses to arousing stimuli.^20^, ^26^ Taken together, these findings are consistent with the known targeting of core cerebral autonomic fronto‐cingulo‐insular circuitry in bvFTD and nfvPPA.^27^, ^28^, ^29^, ^30^ svPPA has also been associated with deficits in afferent interoceptive signal processing.^20^, ^26^, ^31^, ^32^ Altered autonomic reactivity to others’ emotions is a plausible pathophysiological basis for the socio‐emotional symptoms exhibited by these patients. Moreover, autonomic responses and explicit identification of emotions are likely to be separably vulnerable in FTD syndromes.^18^, ^22^, ^27^ However, these issues have not been addressed systematically across the FTD spectrum.

Here, we explored the potential for cardiac emotional reactivity to stratify FTD syndromes. We chose a simple heart rate response metric designed to incorporate both the cardiac orienting response and its potentiation by emotional content, with a view to easy replicability in future studies and potential clinical utility without the need for complex modeling of heart rate patterns. We hypothesized that cardiac modulation would be attenuated in bvFTD and nfvPPA due to degeneration of fronto‐insular networks in these diseases, but relatively preserved (and separable from emotion identification) in syndromes targeting the anterior temporal lobes (svPPA and rtvFTD).^18^, ^20^, ^21^ We further hypothesized that emotion recognition ability but not cardiac reactivity would be associated with semantic knowledge, while cardiac reactivity would correlate with atrophy in components of the central autonomic regulatory network (ACC, insula, OFC).^15^, ^16^, ^24^

## Methods

### Participants

Fifty‐one participants were included in the experiment (mean age 67.6 years (range 51–84), 22 females), comprising 32 patients fulfilling consensus criteria for a syndrome of FTD^33^, ^34^ (10 bvFTD, 6 rtvFTD, 7 svPPA, 9 nfvPPA) recruited via our specialist cognitive disorders clinic, and 19 age‐matched healthy individuals with no history of neurological or psychiatric illness recruited via our departmental research database. No participant had a history of cardiac arrhythmia, and none was taking cardiac rate‐limiting medication. Brain MR imaging supported the syndromic diagnosis in all patients and none had any substantial burden of cerebrovascular disease. In all patients, the syndromic diagnosis was further corroborated in a comprehensive general neuropsychological assessment. Clinical, demographic, and neuropsychological characteristics of all participant groups are summarized in Table 1.

**Table 1:** Demographic, clinical, and neuropsychological characteristics of participant groups

Characteristic	Healthy controls	bvFTD	rtvFTD	svPPA	nfvPPA
Demographic and clinical					
No. (m:f)	19 (8:11)	10 (7:3)	6 (6:0)	7 (5:2)	9 (4:5)
Age (yrs)	68.8 (5.5)	67 (6.3)	63.8 (9.1)	65.9 (7.5)	69.6 (6.5)
Handedness (R:L)	18:1	9:1:0	6:0:0	7:0:0	7:2:0
Education (yrs)	15.5 (2.9)	12.8 (2.5)^c^	18 (3.1)	15.3 (2.8)	15 (2.7)
MMSE (/30)	29.6 (0.6)	24.1 (4.9)^a^	25.3 (4.3)	22.6 (5.8)^a^	23.7 (6.0)^a^
Duration (yrs)	‐	8.2 (5.3)	6.5 (3.5)	4.4 (2.1)	4.6 (2.2)
Mean heart rate	69.5 (10.2)	72.9 (14.2)	71.8 (11.8)	69.7 (5.2)	85.5 (17.1)^a^
Heart rate variance	0.23 (0.7)	0.21 (0.6)	0.05 (0.07)	0.08 (0.08)	0.03 (0.04)
Cardiac reactivity index	1.67 (1.5)	0.54 (0.4)^a^^,^ c	2.42 (1.4)	1.61(1.6)	0.12 (1.1)^a^^,^ c
Emotion recognition (%)	70.5 (9.2)	41.4 (18.9)^a^	40.0 (19.4)^a^	40.2(16.1)^a^	53.8 (18.5)^a^
Neuropsychological					
General intellect					
WASI verbal IQ	125.4 (7.0)	86.2 (23.7)^a^	86.7 (22.2)^a^	78.6(20.4)^a^	79.6 (17.3)^a^
WASI performance IQ	125.1 (9.7)	99.8 (20.2)^a^	106.8 (24.6)	112.3(10.1)	98.8 (21.5)^a^
Episodic memory					
RMT words (/50)	44.7 (3.7)	33.5 (7.9)^a^	34.8 (7.9)^a^	32.7 (6.4)^a^	39.5 (6.6)
RMT faces (/50)	49.3 (0.9)	35.6 (7.5)^a^	37.2 (9.3)^a^	30.3 (6.9)^a^^,^ e	41.4 (9.5)^a^
Camden PAL (/24)	20.3 (3.5)	9.3 (8.2)^a^	12.5 (6.2)	2.7 (4.2)^a^^,^ c ^,^ e	16.3 (7.8)
Executive skills					
WASI Block Design (/71)	46.0 (10.1)	29.9 (17.9)	37.2 (22.1)	41.6 (19.0)	25.1 (19.7)^a^
WASI Matrices (/32)	26.6 (4.1)	17.1 (9.6)^a^	19.0 (9.8)	21.7 (8.5)	17.4 (9.0)^a^
WMS‐R digit span forward (max)	7.1 (1.2)	6.4 (1.3)	6.8 (1.2)	7.0 (1.2)	4.8 (0.8)^a^^,^ c ^,^ d
WMS‐R digit span reverse (max)	5.6 (1.3)	4.2 (1.5)	4.7 (1.4)	5.1 (2.0)	3.0 (0.7)^a^
D‐KEFS Stroop color naming (s)	32.4 (6.4)^e^	49.9 (21.7)^e^	48.8 (21.4)^e^	50.3 (27.9)^e^	87.0 (6.7)
D‐KEFS Stroop word reading (s)	23.5 (5.7)^e^	34.3 (20.9)^e^	38.7 (26.1)^e^	30.9 (19.2)^e^	85.4 (10.3)
D‐KEFS Stroop interference (s)	56.2 (16.9)^b^^,^ e	106.2 (50.7)^e^	98.3 (45.1)^e^	82.7 (50.5)^e^	165.0 (30.1)
Letter fluency (F: total)	18.1 (5.7)	6.8 (4.3)^a^	9.0 (4.7)^a^	9.7 (7.2)^a^	3.5 (1.7)^a^
Category fluency (animals: total)	24.7 (5.9)	12.4 (7.7)^a^	10.3 (2.3)^a^	6.7 (5.4)^a^	8.8 (3.5)^a^
Trails A (s)	32.2 (5.6)^e^	59.3 (35.5)	59.8 (32.9)	47.0 (21.0)	81.7 (48.4)
Trails B (s)	66.1 (20.5)^b^^,^ c ^,^ e	182.5 (87.2)	186.7 (100.4)	133.6 (110.1)	211.1 (94.6)
Language skills					
WASI vocabulary (/80)	72.2 (3.4)	39.9 (23.8)^a^	47.0 (19.1)^a^	34.7 (22.7)^a^	31.7 (13.9)^a^
BPVS (/150)	148.5 (1.1)	112.9 (41.3)^a^	141.8 (7.2)	94.4 (49.4)^a^^,^ c ^,^ e	142.6 (10.1)
GNT (/30)	26.3 (2.4)	9.4 (9.9)^a^	12.5 (10.1)^a^	2.0 (5.3)^a^^,^ c ^,^ e	15.5 (6.6)^a^
Other skills					
GDA (/24)	15.8 (5.4)	7.9 (5.7)^a^	7.5 (6.3)^a^	11.3 (8.3)	5.4 (1.9)^a^
VOSP Object Decision (/20)	19.1 (1.6)	15.0 (3.3)^a^	16.7 (2.3)	15.7 (5.1)	15.3 (4.7)

Mean (standard deviation) scores are shown unless otherwise indicated; maximum scores are shown after tests (in parentheses). BPVS, British Picture Vocabulary Scale;^46^ bvFTD, patient group with behavioral variant frontotemporal dementia; Category fluency for animal category and letter fluency for the letter F in 1 min;^47^ GDA, Graded Difficulty Arithmetic;^48^ GNT, Graded Naming Test;^49^ MMSE, Mini‐Mental State Examination score;^50^ PAL, Paired Associate Learning test^51^; nfvPPA, patient group with nonfluent variant primary progressive aphasia; RMT, Recognition Memory Test;^52^ rtvFTD, patient group with right temporal variant frontotemporal dementia (defined from inspection of individual brain MRI); svPPA, patient group with semantic variant primary progressive aphasia; Stroop D‐KEFS, Delis Kaplan Executive System;^53^ Trails‐making task based on maximum time achievable 2.5 min on task A, 5 min on task B;^54^ VOSP, Visual Object and Spatial Perception Battery;^55^ WAIS‐R, Wechsler Adult Intelligence Scale – Revised;^56^ WASI, Wechsler Abbreviated Scale of Intelligence;^57^ WMS, Wechsler Memory Scale.^58^

a Different from controls.

b Different from bvFTD.

c Different from rtvFTD.

d Different from svPPA.

e Different from nfvPPA (all at significance threshold *P* < 0.05).

### Standard protocol approvals, registrations, and patient consents

The study was approved by the local institutional ethics committee and all participants gave informed consent following Declaration of Helsinki guidelines.

### Stimuli

Videos of emotional facial expressions were taken from the Face and Gesture Recognition Research Network database^35^; these videos are silent recordings of healthy young adults (further details about the stimuli are summarized in Table S1 in Supplementary Material online). These dynamic, naturalistic facial expressions are similar to those encountered in the unregulated social milieu of daily life; we anticipated that such stimuli should induce greater physiological responses than less ecological, static stimuli.^36^ We selected 10 videos (minimizing emotional ambiguity and balancing for sex) to represent each of the “universal emotions” of anger, disgust, fear, happiness, and surprise for a total of 50 trials. We omitted the emotion of sadness, as naturalistic sadness has a more diffuse time course than other emotions, and is therefore less suitable for an analysis of event‐related physiology. Each video stimulus lasted several seconds (mean 4.9 sec; range 4–8 sec), beginning with a neutral facial expression that evolved into an emotional expression. We did not include an “emotionally neutral” facial movement condition; there is currently no dynamic facial “baseline” stimulus widely accepted to be devoid of affective content. For each video, the frame in which each emotional expression began to emerge from the baseline neutral expression was identified manually; the timing of this frame (which occurred between 0.6 and 2.6 sec (mean 1.1 sec) after video onset) was used to align data traces between trials.

Stimuli were presented in randomized order via a notebook computer using Cogent presentation software in MatlabR2012b. On each trial, the participant was asked to identify the emotion by selecting one of the five alternative emotion names. Subjects were unable to provide an answer until after the stimulus had finished playing, and were then able to either select a response by pressing a number key, or pointing out the answer to the tester. The minimum interstimulus interval was 8 sec, and the typical duration of the testing session with cardiac recording was around 20 min. After sitting quietly at rest for at least 5 min, participants were initially familiarized with the stimuli to ensure they all understood the task and were monitored by the experimenter during the test to ensure they were able to comply.

### ECG recording and analysis

ECG was recorded continuously from electrodes over the right clavicle and left iliac crest. ECG data were high‐pass filtered at 0.01 Hz to remove linear drift and establish a baseline from which the time point of each R wave local maximum was determined. Mean heart rate and heart rate variability (variance of RR intervals) during the period of recording were calculated for each participant. A simplified index of cardiac reactivity to viewing facial emotion was derived for each trial as the percentage change in RR interval for three heart beats before and after the onset of each facial expression, to capture both the orienting responses and its potentiation by affective content, using the formula:

([mean of 3 RR intervals after onset] - [mean of 3 RR intervals before onset])×100/mean RR interval

Cardiac reactivity was calculated for each participant for each emotion separately and averaged across all five emotions to provide a measure of overall emotional autonomic reactivity.

The cardiac reactivity index (as defined above) was assessed for each emotion using one‐sample Mann–Whitney *U*‐tests versus zero (no heart rate response) and in a parametric model incorporating both cardiac reactivity and mean heart rate. Between‐group differences were initially assessed using anovas and post hoc *t*‐tests were used to compare groups if a significant overall group effect was shown. For non‐normally distributed data, equivalent nonparametric tests were used (Kruskal–Wallis rank and post hoc Mann–Whitney *U*). Between‐group differences in categorical variables (i.e., sex and handedness) were assessed using chi‐square contingency tests. We used a multiple regression model to test whether any relationship between group membership and cardiac reactivity persisted after covarying for emotion recognition ability and semantic knowledge. A threshold *P* < 0.05 was accepted as the criterion of statistical significance for all group comparisons.

### Brain image acquisition and analysis

For each patient, a sagittal 3‐D magnetization‐prepared rapid‐gradient‐echo T1‐weighted volumetric brain MR sequence (TE/TR/TI 2.9/2200/900 msec, dimensions 256 256 208, voxel volume 1.1^3^ mm) was acquired on a Siemens Trio 3T MRI scanner using a 32‐channel phased‐array head‐coil. Preprocessing of brain images was performed in SPM12 (http://www.fil.ion.ucl.ac.uk/spm) using an optimized protocol.^37^ Normalization, segmentation and modulation of gray and white matter images were carried out using default parameter settings and gray matter images were smoothed using a 6 mm full width‐at‐half‐maximum Gaussian kernel. For each patient, total intracranial volume was calculated by combining gray matter, white matter and cerebrospinal fluid volumes after segmentation of these tissue classes.

In the VBM analysis, associations between regional gray matter volume and both heart rate reactivity and emotion identification performance were assessed in a full factorial model (see Figure S1 in Supplementary Material), looking for an interaction between syndromic group and cardiac reactivity for those patient groups showing altered heart rate reactivity relative to healthy controls and incorporating age, total intracranial volume, and group membership as covariates of no interest. Statistical parametric maps were evaluated at peak voxel threshold *P* < 0.05, after family‐wise error (FWE) correction for multiple voxel‐wise comparisons within prespecified anatomical regions of interest. These regions of interest were defined *a priori* based on the cortical components of the central autonomic control network delineated in the healthy brain,^15^, ^16^ and comprised ACC, insula and OFC as defined using the Harvard‐Oxford Brain Atlas (http://fsl.fmrib.ox.ac.uk/fsl/fslwiki/Atlases).

## Results

### Clinical, behavioral, and heart rate reactivity data

Clinical, behavioral and heart rate reactivity data for the participant groups are summarized in Table 1. The participant groups did not differ in age, sex or handedness; patients and healthy controls did not differ in premorbid educational attainment and the patient groups had similar overall symptom duration (all *P* > 0.05).

Emotion identification was impaired in all syndromic groups relative to the healthy control group (overall group effect *F*_(4)_ = 9.7, *P* < 0.001, η^2^ = 0.459; bvFTD, rtvFTD, svPPA all *P* < 0.001, nfvPPA *P* = 0.01). No differences were found between patient groups. Across the patient cohort, emotion identification score correlated strongly with performance on the British Picture Vocabulary Scale (a standard test of semantic knowledge; *r* = 0.576, *P* < 0.001).

Mean heart rate over the entire recording was higher in the nfvPPA group than in healthy controls (*P* = 0.002). No other differences between groups were identified for mean heart rate. Overall heart rate variability during the recording did not differ between participant groups (*P* = 0.33).

**Figure 1:**
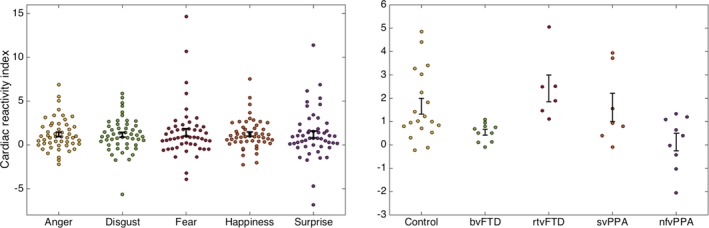
Cardiac reactivity indices by emotion and participant group. Plots show individual participants’ mean cardiac reactivity index (mean percentage change in RR interval, see text) to viewing each of the assessed universal facial emotions (left) and mean overall cardiac reactivity index across viewed emotions, separately for each participant group (right; note change of scale on *y*‐axis). Error bars represent standard error of the mean. bvFTD, patients with behavioral variant frontotemporal dementia; Control, healthy control group; nfvPPA, patients with nonfluent variant primary progressive aphasia; rtvFTD, patients with right temporal variant frontotemporal dementia; svPPA, patients with semantic variant primary progressive aphasia.

Cardiac reactivity indices for all participants are shown for each emotion, and the average over all emotions for each participant group in Figure 1. For the combined participant cohort, an increase in RR interval (cardiac deceleration) was found in response to viewing every emotion (all *P* < 0.001). anova of cardiac reactivity incorporating all emotions showed a main effect of participant group (*P* < 0.001) but not emotion type (*P* = 0.78), nor any interaction of participant group and emotion type (*P* = 0.58). The data for average cardiac reactivity for each subject violated assumptions of homoscedasticity (Levene's test *P* = 0.034) and normality (evident from visualizing a Q‐Q plot of residuals), and were therefore analyzed using nonparametric methods. There was a main effect of participant group on cardiac reactivity averaged over all emotions (Kruskal–Wallis rank test χ^2^_(4)_ = 15.4, *P* = 0.004, estimated η^2^ = 0.273). Post hoc Mann–Whitney *U*‐tests revealed attenuated heart rate responses relative to healthy controls in the bvFTD group (*P* = 0.018) and nfvPPA group (*P* = 0.027) but not the rtvFTD group (*P* = 0.21) or svPPA group (*P* = 0.93). Comparing patient groups, heart rate reactivity was reduced in the bvFTD group (*P* < 0.001) and nfvPPA group (*P* = 0.002) relative to the rtvFTD group; no other differences were identified between patient groups for overall emotion reactivity or reactivity to particular emotions. There was no effect of mean heart rate on cardiac reactivity (*r* = −0.14, *P* = 0.32) and the main effect of participant group on cardiac reactivity persisted after covarying for mean heart rate (*F*_4_ = 3.9, *P* = 0.008). In a combined regression model with cardiac reactivity as the dependent variable, participant group as a fixed factor, and emotion recognition score and British Picture Vocabulary Scale as covariates, the main effect of participant group on cardiac reactivity persisted (*P* = 0.005), but there was no relationship between heart rate reactivity and emotion identification (*P* = 0.79) or general semantic performance (*P* = 0.83).

### Voxel‐based morphometric data

**Figure 2:**
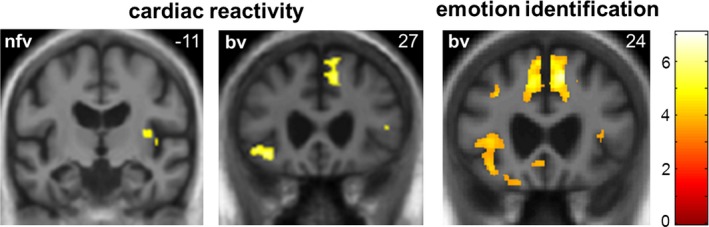
Neuroanatomical correlates of heart rate response to viewing facial emotion and emotion identification in patients. Statistical parametric maps of regional gray matter volume associated with change in RR interval and performance on a facial emotion identification task (derived from a voxel‐based morphometric analysis) are shown for patients with behavioral variant frontotemporal dementia (bv) and nonfluent variant primary progressive aphasia (nfv; these syndromic groups showed an attenuated heart rate response relative to healthy controls). Maps have been overlaid on representative coronal sections of the normalized study‐specific T1‐weighted group mean brain MR image, thresholded at *P* < 0.001 uncorrected over the whole brain for the purpose of display; regional local maxima (see text) were significant at *P* < 0.05_FWE_ corrected for multiple comparisons within prespecified anatomical regions of interest. The MNI coordinate (mm) of the plane of each section is indicated (the right hemisphere is on the right in each case) and the color bar codes T values.

Neuroanatomical associations of heart rate reactivity and emotion identification are summarized in Table 2 and statistical parametric maps of the relevant contrasts are presented in Figure 2, thresholded at *P* < 0.001 uncorrected for display purposes (this threshold was chosen to aid visualization, provide an indication of the overall distribution of change, and avoid suggesting a higher degree of anatomical specificity than is possible with smoothed data). All reported anatomical associations were significant at peak‐level p_FWE_ < 0.05 after correction for multiple voxel‐wise comparisons within the prespecified regions of interest. In the bvFTD group, both reduced heart rate reactivity to viewing facial emotion and reduced emotion identification score were associated with gray matter loss in right dorsal anterior cingulate cortex and left orbitofrontal cortex. Emotion identification in the bvFTD group was additionally associated with gray matter loss in left anterior cingulate cortex and bilateral anterior insula. In the nfvPPA group, reduced heart rate reactivity was associated with gray matter loss in posterior right insula. No gray matter associations of emotion identification were identified in the nfvPPA group at the prescribed threshold.

**Table 2:** Neuroanatomical associations of emotion reactivity and identification in patients

Parameter	Group	Region	Side	Cluster	Peak (mm)			P_FWE_
				(voxels)	*x*	*y*	*z*	
Cardiac reactivity index	bvFTD	Dorsal ACC	R	1040	8	33	33	0.007
		OFC	L	247	−36	27	−12	0.021
							
	nfvPPA	Posterior insula	R	38	36	−10	9	0.044
Emotion identification score	bvFTD	Dorsal ACC	R	852	8	28	45	<0.001
		OFC	L	875	−33	28	0	0.021
		ACC	L	245	−6	45	14	<0.001
		Anterior insula	L	44	−36	−4	15	0.006
		Anterior insula	R	32	40	15	0	0.043

The Table presents gray matter correlates of mean overall cardiac reactivity index (mean percentage change in RR interval, see text) in the bvFTD and nfvPPA groups and emotion identification score in the bvFTD group. Peak coordinates given are in mm in standard MNI space. *P* values are all significant at peak‐level after family‐wise error (FWE) correction for multiple comparisons within prespecified anatomical regions of interest. ACC, anterior cingulate cortex; bvFTD, patient group with behavioral variant frontotemporal dementia; nfvPPA, patient group with nonfluent variant primary progressive aphasia; OFC, orbitofrontal cortex.

## Discussion

Here, we have shown differential impairment of cardiac reactivity to facial emotion across the FTD syndromic spectrum. Cardiac responses to emotional facial expressions, incorporating both orienting and affective components, were attenuated in patients with bvFTD and nfvPPA, relative both to healthy older individuals and to patients with rtvFTD. Patients with svPPA and rtvFTD showed preserved heart rate responses when viewing facial emotions. Across the patient cohort, the degree of heart rate modulation did not correlate with accuracy identifying facial emotions, which was impaired in all syndromic groups. In line with current models of visceral responses to emotion, this work has identified a physiological correlate of reduced emotional responsiveness in FTD, which dissociates from the ability to cognitively (and explicitly) categorize emotions. Our findings further suggest that FTD syndromes are stratified according to the profile of altered autonomic reactivity they exhibit. The findings are consistent with previous work showing reduced autonomic reactivity in bvFTD and nfvPPA^18^, ^26^ and preserved autonomic reactivity in svPPA.^31^ The present work goes further in demonstrating a physiological basis for differentiating subsyndromes within the canonical diagnostic grouping of bvFTD: although a distinct syndrome of rtvFTD has been proposed on neuroanatomical and clinical grounds,^2^, ^3^ these are to a degree arbitrary given the extensive clinico‐anatomical overlap between patients and without mechanistic grounding. Autonomic profiling might establish a principled neurobiological rationale for subclassifying bvFTD, which has long presented nosological difficulties on account of its marked phenotypic and pathological heterogeneity.

Profiles of cardiac reactivity were homogeneous across emotions and did not correlate with explicit emotion identification in our FTD cohort: we propose that autonomic mechanisms govern emotional arousal and intensity (rather than the cognitive categorization of emotions), and are potentially independent of semantic deficits. This interpretation is supported by work in the healthy brain.^10^, ^11^ The subjective experience of emotion is likely to be integral to the internalization of observed emotional states in others during emotional contagion. Our findings therefore provide a candidate neurobiological mechanism for the blunted emotional reactions and loss of empathy that characterize FTD syndromes^38^, ^39^ and amplify previous work linking altered cardiac vagal tone to reduced agreeableness in bvFTD.^24^ Impaired awareness of heartbeat has also previously been demonstrated in FTD^25^, ^32^: taken together with the present findings, this suggests that induction, awareness, and cognitive decoding of embodied emotional responses all contribute to emotional responsiveness and may be separably targeted in FTD syndromes. For example, in svPPA, despite the preserved heart rate response demonstrated here, diminished interpersonal reactivity may be due to reduced afferent processing of these cardiac signals.^32^

This work additionally delineates a neuroanatomical substrate for the differentiated profiles of physiological reactivity and explicit emotion identification in these syndromes. Gray matter associations of heart rate modulation in the bvFTD and nfvPPA groups comprised a predominantly right‐lateralized fronto‐cingulo‐insular “salience” network previously implicated in autonomic regulation in functional neuroimaging studies of healthy individuals^15^, ^40^ and patients with bvFTD.^24^ The components of this network are likely to play hierarchically organized roles in autonomic control, based on predictive integration of internal homeostatic and external affective signals^9^: according to this interoceptive inference formulation, the regulatory network compares incoming afferent information with predicted autonomic states and engages subcortical, modulatory autonomic reflexes in response to prediction errors (unexpected events).^9^ This view emphasizes a reciprocal causality between autonomic responses and subjective emotional states, and suggests mechanisms by which aberrant processing of both afferent and efferent autonomic signals might contribute to reduced emotional reactivity. Posterior insula is the seat of primary interoceptive cortex^41^: noisy processing of cardiac along with other visceral afferent information in this region (as in the nfvPPA group here) would tend to reduce interoceptive sensory precision and therefore lead to reduced prediction errors in response to salient (unexpected) emotional stimuli. Higher stages of the processing hierarchy in ACC and OFC are likely to mediate top‐down control of visceral states by integrating autonomic and cognitive state representations^9^, ^42^; shared neuroanatomical resources for cardiac reactivity and emotion identification in ACC and OFC (as illustrated by the bvFTD group here) would support such integration, as proposed in previous studies of the healthy brain and bvFTD.^19^, ^43^, ^44^ It is also noteworthy that additional gray matter correlates of emotion identification were demonstrated in the bvFTD group (Table 2), suggesting a neuroanatomical substrate for dissociation of affective and cognitive processing over the FTD cohort.

These findings open a window on the pathophysiology of a complex neurodegenerative phenotype. It is of interest that this study employed dynamic emotional stimuli: whether in the domain of vision or sound,^20^, ^31^ stimuli that unfold in time more closely reflect the natural socio‐emotional milieu and may be more adequate for eliciting autonomic responses than the static stimuli that are currently widely used in clinical behavioral experiments. From a clinical perspective, the autonomic profiles reported here constitute simple, quantitative, and readily translatable indices of a behavioral hallmark of FTD (altered emotional responsiveness) that is largely inaccessible to conventional neuropsychological instruments. Indeed, in this study, autonomic metrics proved superior to an emotion identification task in differentiating FTD syndromes, and it is possible that metrics of this kind relate more closely to changes in interpersonal reactivity than does the ability to categorize emotional expressions cognitively. Autonomic indices of this kind warrant further evaluation as disease biomarkers in FTD, particularly with a view to stratifying heterogeneous and poorly demarcated syndromes such as bvFTD and the eventual creation of physiologically informed diagnostic criteria. This will be of considerable practical importance if we are to track disease evolution and the effect of disease modifying therapies dynamically. More immediately, the impaired emotional awareness of patients with FTD is a major determinant of caregiver distress^6^: improved understanding of this symptom would assist counseling and the design of nonpharmacological as well as pharmacological interventions.

This study provides proof‐of‐principle that should direct future work. There is a need for caution in interpreting our findings and, in particular, the practical utility of candidate physiological biomarkers such as cardiac reactivity is yet to be demonstrated. The cohort size here was relatively small, and our findings require corroboration in the wider FTD population. Larger patient cohorts representing a wider range of neurodegenerative pathologies and with additional psychophysiological markers would increase power to detect physiological disease signatures; ultimately, this will require histopathological and molecular correlation. There are successful precedents for large, multi‐center studies of FTD syndromes informed by proof‐of‐principle work in intensively phenotyped patient cohorts.^45^ Experiments to parse the roles played by sympathetic and parasympathetic nervous systems, and the relative contribution of more basic indices of psychophysiological reactivity (such as startle and orienting responses) would further elucidate the neurobiological basis for deficits in FTD. Relatedly, it remains unclear to what extent the cardiac reactivity profiles here are specifically elicited by perceiving facial emotion: in future, this might be resolved by comparing cardiac responses to facial emotional expressions with responses to “neutral” facial movements or emotional vocalizations, or by identifying the core stimulus parameters that convey facial emotion. A number of other factors (e.g., the circadian cycle and concomitant intake of alcohol and stimulants) could in principle modulate cardiac reactivity profiles and these could also be assessed in future studies. Autonomic techniques are potentially well suited for neurodegenerative disease staging and tracking of disease evolution, from the presymptomatic phase in genetic mutation carriers through advanced disease in which neuropsychological assessment may no longer be feasible; however, realizing this potential will require longitudinal analysis of autonomic reactivity indices in different neurodegenerative syndromes. Moreover, these techniques could be readily incorporated in functional neuroimaging studies to define network connectivity.

## Author Contributions

Marshall contributed to the study concept and design, acquisition of data, analysis and interpretation, initial drafting of the manuscript; Hardy contributed to the acquisition of data, analysis and interpretation, critical revision of the manuscript; Allen contributed to the critical revision of the manuscript; Russell contributed to the acquisition of data; Clark contributed to the acquisition of data; Bond contributed to the acquisition of data, critical revision of the manuscript; Dick contributed to the acquisition of data; Brotherhood contributed to the acquisition of data; Rohrer contributed to the study supervision; Kilner contributed to the study concept and design, analysis and interpretation, study supervision; Prof Warren contributed to the study concept and design, analysis and interpretation, critical revision of the manuscript, and study supervision.

## Conflict of Interest

No potential conflicts of interest are identified.

## Supporting information


**Figure S1.** The figure shows the SPM design matrix for the full factorial model used in the voxel‐based morphometry analysis.
**Table S1.** The table presents gender balance and duration for video stimuli selected from the FG‐NET database for presentation in the experiment.
