# Supplementary material for: Cardiac responses to viewing facial emotion differentiate frontotemporal dementias
Source: Ann Clin Transl Neurol. 2018 Apr 14;5(6):687–96. doi: 10.1002/acn3.563 (PMC5989744; doi:10.1002/acn3.563)
Supplement: Supplementary file 1 — Figure S1. The figure shows the SPM design matrix for the full factorial model used in the voxel‐based morphometry analysis. Table S1. The table presents gender balance and duration for video stimuli selected from the FG‐NET database for presentation in the experiment. [file ACN3-5-687-s001.docx]

**Supplementary Material:**

**Cardiac responses to viewing facial emotion differentiate frontotemporal dementias by CR Marshall et al.**

| Emotion | Male:Female | Mean duration (s) |
| --- | --- | --- |
| Anger | 4:6 | 4.0 |
| Disgust | 5:5 | 6.0 |
| Fear | 5:5 | 6.2 |
| Happiness | 6:4 | 4.4 |
| Surprise | 6:4 | 4.1 |
| Overall | 26:24 | 4.9 |

**Table S1.** Gender balance, duration and exemplars for video stimuli selected from the FG-NET database for presentation in the experiment.


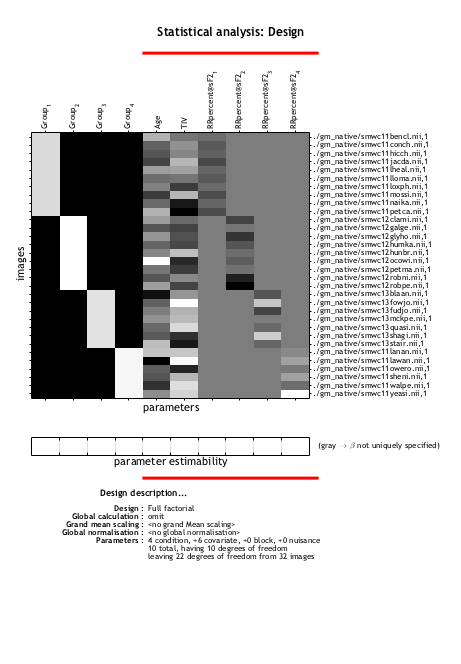


**Figure S1.** SPM design matrix for the full factorial model used in the voxel based morphometry analysis. Contrasts for associations of the parameter of interest within each syndromic group were defined as follows:

bvFTD: 0 0 0 0 0 0 1 0 0 0

nfvPPA: 0 0 0 0 0 0 0 1 0 0

svPPA: 0 0 0 0 0 0 0 0 1 0

rtvFTD: 0 0 0 0 0 0 0 0 0 1
